# Domains of Access for Interventions Addressing Intimate Partner Violence Among Latina Immigrants: Protocol for a Systematic Review

**DOI:** 10.2196/76996

**Published:** 2025-09-11

**Authors:** Erika La Frano, Jamie Conklin, Moses Okumu

**Affiliations:** 1 School of Social Work University of Illinois Urbana-Champaign Urbana, CA United States; 2 Health Sciences Library University of North Carolina at Chapel Hill Chapel Hill, NC United States

**Keywords:** interventions, intimate partner violence, Latina, access to care, systematic review protocol

## Abstract

**Background:**

Latina immigrants in the United States face barriers to accessing information and support for intimate partner violence (IPV) due to language and cultural differences, a lack of legal awareness, social isolation, and financial issues. Despite numerous interventions, research lacks insight into how these programs address access barriers and target IPV-related risk and protective factors.

**Objective:**

This systematic review aims to (1) identify intervention studies conducted with Latina immigrants in reducing IPV outcomes (ie, sexual and physical violence and gender-based violence–related knowledge and attitudes), (2) evaluate the extent to which the implementation of these IPV interventions aligns with the access to care framework, and (3) establish whether the extent of IPV intervention alignment with the access to care framework has any significant impact on effectiveness.

**Methods:**

A systematic review will be conducted using the PRISMA-P (Preferred Reporting Items for Systematic Review and Meta-analysis Protocols) checklist. The research will proceed iteratively among authors. The team, including a librarian, developed a search strategy and searched 7 databases, namely, APA PsycInfo, CINAHL Plus with Full Text, PubMed, Scopus, Social Work Abstracts, GenderWatch, and Sociological Abstracts, from inception to June 9, 2025. Two reviewers independently conducted a double selection of titles, abstracts, and full texts. Disagreements were resolved by a third reviewer. Data extraction will be performed by 2 reviewers and validated by a senior researcher. We will develop an intervention characteristics form for data extraction and analyze the study quality using the modified Methodological Quality Rating Scale. To assess alignment with access implementation standards, we will operationalize the access to care framework by creating the Domains of IPV Service Access Rating Scale to evaluate how interventions address access barriers and determine whether addressing these barriers increases intervention effectiveness.

**Results:**

The search strategy and literature review were finalized in June 2025. A total of 943 references were identified after duplicates were removed, of which 182 will be reviewed in full text. A follow up search will be performed prior to submission. The publication is anticipated for October 2025.

**Conclusions:**

This review will provide a comprehensive synthesis of strategies to address access barriers in IPV interventions for Latina immigrants, informing future practice and research to enhance equitable access to support and resources.

**Trial Registration:**

PROSPERO 42024622171; https://www.crd.york.ac.uk/PROSPERO/view/CRD42024622171

**International Registered Report Identifier (IRRID):**

DERR1-10.2196/76996

## Introduction

### Background

Intimate partner violence (IPV) constitutes a significant public health crisis, disproportionately impacting women in the United States and globally. IPV encompasses physical, sexual, or psychological harm, including stalking and coercive tactics, inflicted by a current or former intimate partner [1]. It is estimated that 1 in 3 women experience IPV [2]. The repercussions of IPV are severe, resulting in an elevated risk of homicide, physical injuries, chronic pain, reproductive health issues, adverse pregnancy outcomes, substance use disorders, and mental health conditions such as posttraumatic stress disorder, depression, and suicidality [3-6]. Within the United States, Latino populations, the largest immigrant group, encounter unique sociocultural and structural challenges that exacerbate their vulnerability to IPV and restrict their access to support [7]. Latinas who have experienced IPV report higher levels of poor physical health and psychological distress than non-Latina women with similar experiences [8,9]. They also face a distinct set of risk factors and barriers when seeking assistance after IPV, including limited legal protection, cultural stigma, and fear of authorities or deportation [10-12]. This underscores an urgent need for evidence-based interdisciplinary responses to guide organizations and service systems in identifying and overcoming barriers to accessing resources and support that are specifically tailored for Latino immigrant populations.

Ensuring access to essential health, mental health, social, and legal services is vital for the recovery and well-being of IPV survivors. However, Latina immigrants face numerous intersecting barriers such as structural challenges (eg, service availability), logistical concerns (eg, transportation and operating hours), financial constraints (eg, costs and insurance), and sociocultural factors (eg, acceptability and language compatibility) that impede their ability to obtain timely, culturally appropriate, and effective assistance [13-15]. Latina survivors use formal services, including health and social services, at significantly lower rates than other IPV survivors [16,17], and Latinas with undocumented status sought formal support services at even lower rates [11]. Research studies have consistently identified factors deterring Latina immigrants from disclosing abuse, including fear of separation from their children, limited information about their legal rights, limited knowledge about community resources, lack of English proficiency, social isolation, and limited access to health care [18,19]. Similarly, in a qualitative metasynthesis of 47 articles over 20 years, Hulley et al [20] found that immigrant women face challenges arising from institutional barriers, immigration status, cultural differences, and insufficient diversity among service providers who speak the language and understand the lived experiences of those they serve.

To better address these challenges, the Domains of Access framework, encompassing availability, accessibility, accommodation, affordability, and acceptability, offers a useful lens for analyzing Latina immigrants' interactions with service systems in the United States. [21]. Within the context of IPV, availability refers to the presence of services and providers capable of addressing the concerns of Latina immigrant women. Yet, many communities, especially rural or underserved areas, experience ongoing shortages of providers, a lack of bilingual staff, and limited cultural understanding of how to address this issue [15,20,22,23]. The accommodation domain involves flexibility around service delivery to meet individual needs, including hours of operation, walk-in hours, literacy levels, and linguistic requirements. Immigrants often work in sectors with limited flexibility for scheduling appointments with providers or advocates [24,25]. Once a survivor receives services, linguistic and cultural alignment with providers facilitates abuse disclosure and improves resource access for immigrant women [26,27]. The affordability domain includes access to health insurance and costs such as transportation and childcare [13,24]. A California study found that immigrant Latinos had poorer health care access than US-born Latinos, with undocumented immigrants accessing fewer resources due to costs or qualification barriers [18]. This results in fewer preventive care visits and missed opportunities for IPV screening. The acceptability domain concerns the relationship between organizations providing support and participants’ comfort in discussing IPV. For instance, in a systematic review investigating effective interventions to address IPV among Latinas, Alvarez et al [28] found that the most effective interventions were gender-specific, culturally tailored, delivered in a group format, and developed collaboratively with the participants. Lastly, accessibility refers to the survivor’s ability to reach and understand the nature of services being offered. Limited public transportation, lack of language and information delivery that accommodate various literacy levels, geographic isolation, and abuser restrictions further constrain immigrant survivors’ ability to access resources and support [25,29,30]. Given these layered barriers, research that identifies and evaluates effective strategies to reduce access gaps is urgently needed.

While the Domains of Access framework provides a valuable lens for understanding the multilevel barriers Latina immigrants face in seeking IPV services, no systematic review has yet applied this model to the issue of violence and service access in this population. This gap has resulted in a fragmented evidence base, limiting the ability of practitioners and policymakers to design interventions that fully account for the complexities of access. Nonetheless, there is growing recognition of the unique needs of Latina immigrants, and targeted multicomponent interventions have begun to emerge addressing information and language requirements, financial constraints, and contextual factors that impede engagement [31]. Although individual interventions have demonstrated promise in supporting immigrant IPV survivors [32-35], this proposed study will be the first to synthesize the evidence on interventions that explicitly address access barriers for Latina immigrants within a comprehensive theoretical framework.

### Objectives

This protocol outlines the methodology for a systematic review to map and synthesize evidence on interventions for IPV among Latina immigrants in the United States and their service access using the Domain of Access framework. The objectives include (1) identifying intervention studies conducted with Latina immigrants to reduce IPV outcomes (ie, sexual and physical violence and gender-based violence–related knowledge and attitudes), (2) evaluating the extent to which the implementation of these IPV interventions aligns with the Domains of IPV Service Access, and (3) establishing whether the extent of IPV intervention alignment with the Domains of IPV Service Access has any significant impact in terms of effectiveness. The review outcomes will inform the development of equitable interventions, practices, and policies for Latina immigrant women experiencing violence.

## Methods

### Reporting Standards 

A systematic review will be conducted by a research team in accordance with the PRISMA-P (Preferred Reporting Items for Systematic Review and Meta-analysis Protocols) checklist [36].

### Search Strategy

The research strategy was developed by a research team in collaboration with a health sciences librarian. The research was conducted in the following databases from their inception until the final search date of June 9, 2025: APA PsycInfo (EBSCOhost), CINAHL Plus with Full Text (EBSCOhost), PubMed, Scopus, Social Work Abstracts (EBSCOhost), Sociological Abstracts (ProQuest), and GenderWatch (ProQuest). The search terms based on a combination of 4 key concepts: ethnic identity, migration, relationship context, and violence and abuse. The complete and reproducible search strategy for all databases is provided in the appendix. The search results were imported into EndNote X9, where duplicates were removed. All unique references were imported into Covidence (Veritas Health Innovation). Two reviewers then proceeded individually and independently to conduct a double selection of the titles, abstracts, and full texts. A third reviewer resolved any disagreements between the reviewers. Data extraction will be performed by a reviewer and validated by a senior researcher.

### Eligibility Criteria

The eligibility criteria will be based on the PICOS (population, intervention, comparison, outcomes, and study design) model [37] and are presented in Table 1. To be included in the review, studies should target Latino immigrant adults and youth in the United States. In addition, all studies on interventions related to IPV, domestic violence, or related forms of violence (eg, dating violence) will be included in the review. Studies with or without a control group will be included in this review. As for the types of studies, we included those with a quantitative component that included measures of targeted outcomes to determine effectiveness (ie, surveys and assessments). This systematic review will consider only studies published in English or Spanish. In addition, studies must have been published as peer-reviewed empirical studies; therefore, reviews, conference proceedings, study protocols, and editorials were excluded. We excluded non-Latina immigrant samples to highlight programs that aimed to address the complexities of immigrant status and IPV, including legal status, social isolation, language, and culture.

**Table 1:** Eligibility criteria based on the PICOS (population, intervention, comparison, outcomes, and study design) model.

PICOS	Criteria
Population (P)	Latina or Hispanic immigrant women in the United States who have experienced IPV^a^.
Intervention (I)	IPV prevention or intervention programs, including culturally tailored, trauma-informed, or community-based approaches.
Comparative (C)	Standard IPV services without cultural tailoring, alternative programs, or no intervention.
Outcomes (O)	The following elements will be examined: Categories: Level of intervention: individual, group, or mixed. Mode of design: theory, empirical evidence, or none. Targeted outcomes: primary and secondary outcomes, operational definitions, and measurement tools (ie, mental health, awareness about IPV and resources, and changes in self-esteem). Characteristics of interventions: intervention name, setting, and target population, Results and success factors of interventions: acceptability of services (ie, retention) and mitigation of access barriers.
Study design (S)	All quantitative empirical or mixed method studies without or without a control group will be included.

^a^IPV: intimate partner violence.

### Study Selection and Extraction

**Figure 1:**
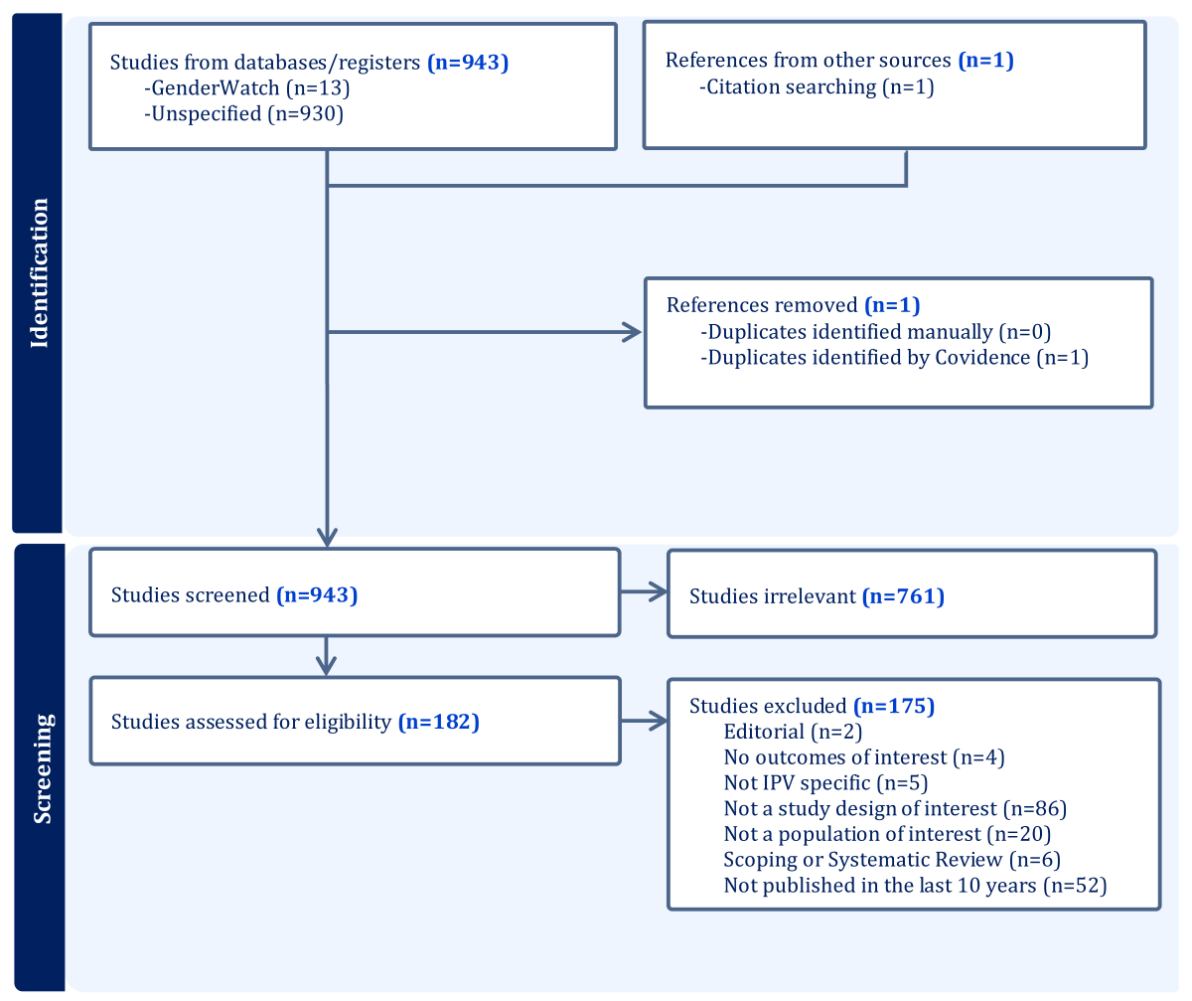
PRISMA flow diagram.

The studies will be selected using Covidence [38]. Reviewers from the research team will independently assess the titles and abstracts, followed by the full texts of the relevant studies. Figure 1 presents a flowchart depicting the search and screening process. After study selection, data will be gathered by a reviewer and verified by a senior team member using an extraction grid created and tested by the research team. A third reviewer will resolve any conflicts or disagreements that arise. We will assess interrater reliability using Cohen κ to measure agreement prior to reconciliation. For quantitative data, reconciliation will include a comparison of the extracted values with the original source to verify accuracy.

### Data Synthesis and Analysis

We will use narrative synthesis to synthesize the data. We will provide a descriptive synthesis of the results of all included studies. A narrative summary of the main results will be produced to gather comprehensive information from each included study systematically. The extracted data will encompass key characteristics of the intervention (eg, intervention name, setting, delivery format, target population, and sample size), study design and methodological features (eg, design type, theoretical framework, intervention duration, and follow-up period), intervention components (eg, targeted risk and protective factors and cultural or contextual adaptations), and outcomes (primary and secondary outcomes, operational definitions, and measurement tools). Additionally, we will present the results related to the identification and mitigation of access barriers. Two reviewers will independently extract the data, with any discrepancies resolved through discussion or consultation with a third reviewer if necessary. All extracted data will be organized into structured tables to facilitate detailed comparisons and syntheses across the studies. The analysis will involve the tabulation and systematic review of variables pertinent to the study objectives, ensuring the consistency, transparency, and reproducibility of the review process.

### Methodological Quality Assessment

Two reviewers will assess the methodological rigor using a modified version of Miller et al’s [39] Methodological Quality Rating Scale, as described by Nyoni et al [40] (Table 2). This adaptation extends the original 14-item Methodological Quality Rating Scale by adding 3 items (theoretical basis, intervention intensity, and multiple outcome measures), removing 2 items (contact and collaterals), merging dropout and attrition into 1 item, and expanding scoring for study design and multisite assessment. The revised scale allows a maximum score of 24, ensuring a systematic evaluation of methodological rigor across the included studies. Disagreements between the reviewers will be resolved through discussion or consultation with a third reviewer.

**Table 2:** A Methodological Quality Rating Scale.

Methodological criteria	Rating (points)
Study design	3 – Longitudinal randomized control trial 2 – Mixed methods 1 – Open pilot trial 0 – Single group (pretest or posttest)
Theoretical foundations	1 – Intervention theory reported 0 – Intervention theory not reported
Quality control	2 – Standardized by manual procedures, specific training, and quality control measures 1 – Standardized by manual procedure or specific training 0 – No standardization reported
Follow-up rate	2 – Equal or greater than the intervention phase 1 – 70% to 99% of the intervention phase 0 – Less than 70% of the intervention phase
Follow-up length	2 – 12 months or longer 1 – 6 to 11 months 0 – Less than 6 months
Measures	1 – Reliability and validity measures reported 0 – Reliability and validity measures not reported
Multimethod measures	3 – Four or more IPV and associated risk or protective factors 2 – Three measures of IPV and risk or protective factors 1 – Any two of the measures 0 – One measure
Objective verification	1 – Objective verification reported 0 – Objective verification not reported
Intervention intensity	2 – Treatment group receives three or more components 1 – Treatment group receives two components 0 – Treatment group receives one component
Dropouts and attrition	1 – Dropouts enumerated or discussed 0 – Dropouts not enumerated or discussed
Blind follow-up	1 – Follow-up by a person blind to participants’ condition 0 – Nonblinded or no follow-up
Analysis	1 – Appropriate statistical analysis (between-group differences, control for baseline differences) 0 – No statistical analysis conducted
Multisite	3 – Parallel replications within the United States 2 – Within the state 1 – Within the same community 0 – No replication
Generalizability	1 – Generalizability discussed (eg, sample characteristics, site, and curriculum) 0 – Not discussed

### Assessment of Study Compliance With Domains of IPV Service Access

To evaluate how IPV intervention studies among Latina immigrants in the United States address access barriers, we will develop a Domains of IPV Service Access Rating Scale (DISARS). This 5-item scale will assess the extent to which interventions incorporate (1) availability, (2) accommodation, (3) affordability, (4) acceptability, and (5) accessibility. In developing DISARS, we will draw upon prior empirical conceptualizations [40] (refer to Table 3). For each intervention, we will rate performance across the items and calculate a composite DISARS score to quantify adherence to access-oriented implementation standards. The DISARS will also allow us to explore whether greater alignment with the Domains of IPV Service Access is associated with enhanced intervention effectiveness.

**Table 3:** The operationalization of the concepts used for intervention study appraisal.

Domain	Definition	Operationalization of the concept
Availability	The presence of services to address IPV^a^ in rural or urban areas with a large concentration of Latinos.	Services or resources to screen and support participants are present in clinics or community centers. Public transportation services are present in the area.
Accommodation	The presence of sensitivity around issues of immigration status and other elements that may impact women’s ability to access interventions or other forms of support.	Information and resources are delivered in the languages spoken by Latina immigrants. The hours of operation account for work schedules with little flexibility. Immigration status–related concerns were addressed.
Affordability	The relationship between the cost of accessing the intervention and the women’s ability to pay for it.	Interventions and services were made available for participants regardless of health insurance. Transportation is provided and childcare is offered. Information about discounted or free phones and internet was made available.
Acceptability	The relationships between the approach to delivering the intervention that address IPV and the participants’ attitudes toward and comfort with the content.	The mode of delivery is acceptable for this population (ie, group format). Community health workers provide input into the design and mode of delivery. The curriculum is delivered alongside other culturally relevant activities.
Accessibility	Information about IPV and related resources is made available and can be understood by the participants	Information is available to different literacy levels. Linkage to legal or other social services is provided as part of the intervention. The intervention is situated in a location familiar to the participants and accessible within walking distance.

^a^IPV: intimate partner violence.

### Ethical Considerations

Ethical approval is not necessary because this is a protocol for a systematic review. The results will be shared through publications in peer-reviewed journals and presentations at conferences.

## Results

The search strategy and literature review were finalized in June 2025. A total of 943 references were identified after duplicates were removed, of which 18 will be reviewed in full text. The publication of this systematic review is scheduled for September 2025.

## Discussion

### The Main Contributions of This Systematic Review

This systematic review protocol is designed to address a critical gap in the literature on how interventions for Latina immigrants experiencing IPV address barriers to service access across the Domains of Access framework. While previous reviews have examined IPV prevalence among Latinas [41], key components of effective interventions [28], the effectiveness of culturally tailored interventions [42], the effectiveness of group-format programs [43], and the intersection of IPV and immigration status [44], there remains a limited insight into how interventions explicitly operationalize and address access barriers, particularly in the domains of availability, acceptability, accessibility, affordability, and accommodation. Applying the Domains of Access model enables a comprehensive exploration of barriers and facilitators that extend beyond individual or cultural determinants. This theoretically informed approach has the potential of revealing the structural, organizational, and interpersonal dynamics that often intersect to either enable or hinder service access, especially among marginalized and vulnerable populations such as Latina immigrants. Additionally, this review’s focus on Latina immigrants in the US addresses an important but underserved area where cultural, linguistic, legal, and sociostructural complexities are frequently overlooked in mainstream discourses and research on violence and service access [31]. Taken together, the findings of this review may be critical for developing more targeted multicomponent interventions that respond to the specific needs of individuals facing one or more of these challenges.

### Potential Impact and Future Directions

This systematic review will influence violence prevention and service accessibility for Latina immigrants in the United States. Using the Domains of Access framework, it analyzes barriers and facilitators affecting survivors’ experiences, providing practitioners, policymakers, and researchers with insights into strategies that have shown promise in improving service access for Latina immigrants experiencing IPV. Prior research suggests that culturally and linguistically tailored group-format programs developed collaboratively with the target population can enhance service acceptability and engagement [28,42,43]. Building on these insights, this review not only synthesizes existing strategies but also highlights critical gaps, including the limited availability of interventions addressing social and linguistic isolation [32], cultural factors [45-47], and financial burdens extending beyond health insurance and transportation [31]. By applying a domains-based approach, this review also establishes standards for future systematic reviews in migration, violence, and public health research, fostering methodological innovation and enhancing transparency.

Identifying the key elements that facilitate broader access to IPV-related resources and support contributes to the development of effective and innovative interventions that meet the needs of this population. The literature shows significant variability in the design, integration of culturally specific elements, and focus on prevention versus response among interventions targeting Latinas, along with a need for research that emphasizes cultural strengths as well as structural barriers [48]. The wide variability in intervention design could be explained by the variety of measurement tools, recruitment strategies, integration of mental health services, and geographic settings. These elements could also explain why some interventions are more effective than others in achieving their goals. Therefore, there is a need to comprehensively understand and highlight effective interventions that engage Latina immigrant survivors of IPV and the factors that these interventions address to promote more equitable access.

Although this review aims to provide a thorough overview, it is important to acknowledge several methodological limitations. First, the search was restricted to studies published in English and Spanish, which may have excluded relevant research in other languages. Second, relying mainly on major academic databases might overlook studies indexed in regional or less conventional sources, limiting comprehensiveness. Finally, since the focus is on Latina immigrant women, the findings may not fully reflect the experiences of Latino men, nonbinary individuals, or other underrepresented groups. Recognizing these limitations promotes transparency, situates the findings within their proper context, and emphasizes that this review should be understood as part of an expanding and evolving evidence base.

### Conclusions

The outcomes of this systematic review will provide a comprehensive overview of effective interventions that mitigate access barriers among Latina immigrants. They will elucidate the characteristics of intervention studies that focus on IPV-related risk and protective factors while enhancing access to information and support. The findings will constitute a valuable repository of knowledge and evidence, illustrating effective strategies for rigorous and methodologically diverse interventions, particularly in light of a rapidly evolving immigration landscape.

## Appendix

Multimedia Appendix 1 Search strategy report.

## Abbreviations

DISARS: Domains of IPV Service Access Rating Scale
IPV: intimate partner violence
PICOS: population, intervention, comparison, outcomes, and study design
PRISMA: Preferred Reporting Items for Systematic Review and Meta-Analyses
PRISMA-P: Preferred Reporting Items for Systematic Review and Meta-Analyses Protocol

## Data Availability

Data related to the search strategy for this review are provided in Multimedia Appendix 1. All data generated or analyzed, including the PRISMA (Preferred Reporting Items for Systematic Review and Meta-Analyses) flowchart, will be provided as multimedia appendices. The databases compiled during data extraction are available upon reasonable request.
