# Supplementary material for: Domains of Access for Interventions Addressing Intimate Partner Violence Among Latina Immigrants: Protocol for a Systematic Review
Source: JMIR Res Protoc. 2025 Sep 11;14:e76996. doi: 10.2196/76996 (PMC12464507; doi:10.2196/76996)
Supplement: Multimedia Appendix 1 [file resprot_v14i1e76996_app1.docx]

**Search Strategy Report:**

Date: 6/09/2025

Total # of References: 2259

# of Duplicates Removed: 1317

Total # of References to Screen: 942

Database: PubMed

| Set # |  | Results |
| --- | --- | --- |
| 1 | "Hispanic or Latino"[Mesh] OR Hispanic[tiab] OR Hispanics[tiab] OR Latina[tiab] OR Latinas[tiab] OR Latino[tiab] OR Latinos[tiab] OR Latine[tiab] OR Latinx[tiab] OR Chicana[tiab] OR Chicanas[tiab] OR Chicano[tiab] OR Chicanos[tiab] OR “Cuban American”[tiab] OR “Cuban Americans”[tiab] OR “Mexican American”[tiab] OR “Mexican Americans”[tiab] OR “Puerto Rican”[tiab] OR “Puerto Ricans”[tiab] OR “Spanish American”[tiab] OR “Spanish Americans”[tiab] OR “US Latin American”[tiab] OR “US Latin Americans”[tiab] |  |
| 2 | "Emigrants and Immigrants"[Mesh] OR "Emigration and Immigration"[Mesh] OR "Transients and Migrants"[Mesh] OR "Refugees"[Mesh] OR "Acculturation"[Mesh] OR migrant[tiab] OR migrants[tiab] OR immigrant[tiab] OR immigrants[tiab] OR immigration[tiab] OR migration[tiab] OR immigrated[tiab] OR migrated[tiab] OR immigrating[tiab] OR migrating[tiab] OR immigrate[tiab] OR migrate[tiab] OR immigrates[tiab] OR migrates[tiab] OR refugee[tiab] OR refugees[tiab] OR asylum[tiab] OR displaced[tiab] OR acculturation[tiab] OR acculturate[tiab] OR acculturates[tiab] OR acculturated[tiab] OR acculturating[tiab] OR assimilation[tiab] OR assimilate[tiab] OR assimilates[tiab] OR assimilated[tiab] OR assimilating[tiab] |  |
| 3 | "Family"[Mesh] OR "Marriage"[Mesh] OR "Sexual Partners"[Mesh] OR "Battered Women"[Mesh] OR domestic[tiab] OR family[tiab] OR families[tiab] OR partner[tiab] OR partners[tiab] OR spouse[tiab] OR spouses[tiab] OR spousal[tiab] OR marriage[tiab] OR married[tiab] OR marital[tiab] OR couple[tiab] OR couples[tiab] OR husband[tiab] OR husbands[tiab] OR wife[tiab] OR wives[tiab] OR dating[tiab] OR date[tiab] OR boyfriend[tiab] OR boyfriends[tiab] OR girlfriend[tiab] OR girlfriends[tiab] OR interpersonal[tiab] OR “gender based”[tiab] OR gender-based[tiab] |  |
| 4 | "Violence"[Mesh] OR "Aggression"[Mesh] OR "Harassment, Non-Sexual"[Mesh] OR "Sexual Harassment"[Mesh] OR "Incivility"[Mesh] OR "Stalking"[Mesh] OR "Emotional Abuse"[Mesh] OR violence[tiab] OR cyberviolence[tiab] OR violent[tiab] OR abuse[tiab] OR abused[tiab] OR abuses[tiab] OR abuser[tiab] OR abusers[tiab] OR cyberabuse[tiab] OR cyberabuses[tiab] OR cyberabuser[tiab] OR cyberabusers[tiab] OR assault[tiab] OR assaulted[tiab] OR assaults[tiab] OR assaulter[tiab] OR assaulters[tiab] OR cyberassault[tiab] OR cyberassaulted[tiab] OR cyberassaults[tiab] OR cyberassaulter[tiab] OR cyberassaulters[tiab] OR assailant[tiab] OR assailants[tiab] OR battery[tiab] OR battered[tiab] OR batterer[tiab] OR batterers[tiab] OR beat[tiab] OR beaten[tiab] OR beats[tiab] OR beating[tiab] OR perpetrator[tiab] OR perpetrators[tiab] OR perpetration[tiab] OR perpetrating[tiab] OR perpetrated[tiab] OR perpetrate[tiab] OR perpetrates[tiab] OR rape[tiab] OR raped[tiab] OR rapes[tiab] OR rapist[tiab] OR rapists[tiab] OR mistreatment[tiab] OR mistreated[tiab] OR mistreatments[tiab] OR molestation[tiab] OR molested[tiab] OR molestations[tiab] OR molester[tiab] OR molesters[tiab] OR attacked[tiab] OR attacks[tiab] OR attack[tiab] OR attacker[tiab] OR attackers[tiab] OR cyberattacked[tiab] OR cyberattacks[tiab] OR cyberattack[tiab] OR cyberattacker[tiab] OR cyberattackers[tiab] OR coercive[tiab] OR coercion[tiab] OR aggression[tiab] OR aggressive[tiab] OR aggressor[tiab] OR aggressors[tiab] OR cyberaggression[tiab] OR harassment[tiab] OR harassments[tiab] OR harassed[tiab] OR harass[tiab] OR harasses[tiab] OR harassing[tiab] OR stalking[tiab] OR stalked[tiab] OR stalker[tiab] OR stalkers[tiab] OR cyberstalking[tiab] OR cyberstalked[tiab] OR cyberstalker[tiab] OR cyberstalkers[tiab] OR victimization[tiab] OR victimized[tiab] OR victimize[tiab] OR victimizes[tiab] OR victimizing[tiab] OR cybervictimization[tiab] OR cybervictimized[tiab] OR cybervictimize[tiab] OR cybervictimizes[tiab] OR cybervictimizing[tiab] OR terrorism[tiab] OR cyberterrorism[tiab] OR bullying[tiab] OR bullied[tiab] OR bully[tiab] OR bullies[tiab] OR cyberbullying[tiab] OR cyberbullied[tiab] OR cyberbully[tiab] OR cyberbullies[tiab] OR blackmail[tiab] OR blackmailed[tiab] OR blackmails[tiab] OR blackmailing[tiab] OR bribery[tiab] OR bribe[tiab] OR bribes[tiab] OR bribed[tiab] OR bribing[tiab] OR incivility[tiab] OR cyberincivility[tiab] OR defamation[tiab] OR defames[tiab] OR defamed[tiab] OR defaming[tiab] OR slander[tiab] OR slanderous[tiab] OR exploitation[tiab] OR exploited[tiab] OR exploit[tiab] OR exploits[tiab] OR exploiting[tiab] OR sexploitation[tiab] OR “hate speech”[tiab] OR sextortion[tiab] OR “sexual solicitation”[tiab] OR “unwanted sex”[tiab] OR “unwanted sexual”[tiab] OR extortion[tiab] OR doxxing[tiab] OR hacking[tiab] OR “online impersonation”[tiab] OR “false identity”[tiab] OR “false identities”[tiab] OR “non-consensual sharing”[tiab] OR shallowfake[tiab] OR shallowfakes[tiab] OR deepfake[tiab] OR deepfakes[tiab] OR “predatory behavior”[tiab] OR “predatory behaviors”[tiab] OR predator[tiab] OR predators[tiab] OR predatorial[tiab] OR cyberpredator[tiab] OR cyberpredators[tiab] |  |
| 5 | #1 AND #2 AND #3 AND #4 | 432 |

Database: APA PsycInfo (EBSCO*host*)

| Set # |  | Results |
| --- | --- | --- |
| 1 | DE "Latinos/Latinas" OR DE "Mexican Americans" OR TI (Hispanic OR Hispanics OR Latina OR Latinas OR Latino OR Latinos OR Latine OR Latinx OR Chicana OR Chicanas OR Chicano OR Chicanos OR “Cuban American” OR “Cuban Americans” OR “Mexican American” OR “Mexican Americans” OR “Puerto Rican” OR “Puerto Ricans” OR “Spanish American” OR “Spanish Americans” OR “US Latin American” OR “US Latin Americans”) OR AB (Hispanic OR Hispanics OR Latina OR Latinas OR Latino OR Latinos OR Latine OR Latinx OR Chicana OR Chicanas OR Chicano OR Chicanos OR “Cuban American” OR “Cuban Americans” OR “Mexican American” OR “Mexican Americans” OR “Puerto Rican” OR “Puerto Ricans” OR “Spanish American” OR “Spanish Americans” OR “US Latin American” OR “US Latin Americans”) |  |
| 2 | DE "Immigration" OR DE "Undocumented Immigration" OR DE "Refugees" OR DE "Migrant Workers" OR DE "Migrant Farm Workers" OR DE "Acculturation" OR TI (migrant OR migrants OR immigrant OR immigrants OR immigration OR migration OR immigrated OR migrated OR immigrating OR migrating OR immigrate OR migrate OR immigrates OR migrates OR refugee OR refugees OR asylum OR displaced OR acculturation OR acculturate OR acculturates OR acculturated OR acculturating OR assimilation OR assimilate OR assimilates OR assimilated OR assimilating) OR AB (migrant OR migrants OR immigrant OR immigrants OR immigration OR migration OR immigrated OR migrated OR immigrating OR migrating OR immigrate OR migrate OR immigrates OR migrates OR refugee OR refugees OR asylum OR displaced OR acculturation OR acculturate OR acculturates OR acculturated OR acculturating OR assimilation OR assimilate OR assimilates OR assimilated OR assimilating) |  |
| 3 | DE "Family" OR DE "Biological Family" OR DE "Dysfunctional Family" OR DE "Extended Family" OR DE "Family Background" OR DE "Family History" OR DE "Family Members" OR DE "Family of Origin" OR DE "Family Relations" OR DE "Family Resemblance" OR DE "Family Structure" OR DE "Family Work Relationship" OR DE "Interethnic Family" OR DE "Interracial Family" OR DE "Marriage" OR DE "Military Families" OR DE "Nepotism" OR DE "Nuclear Family" OR DE "Offspring" OR DE "Stepfamily" OR DE "Battered Females" OR DE "Partners" OR DE "Sexual Partners" OR TI (domestic OR family OR families OR partner OR partners OR spouse OR spouses OR spousal OR marriage OR married OR marital OR couple OR couples OR husband OR husbands OR wife OR wives OR dating OR date OR boyfriend OR boyfriends OR girlfriend OR girlfriends OR interpersonal OR “gender based” OR gender-based) OR AB (domestic OR family OR families OR partner OR partners OR spouse OR spouses OR spousal OR marriage OR married OR marital OR couple OR couples OR husband OR husbands OR wife OR wives OR dating OR date OR boyfriend OR boyfriends OR girlfriend OR girlfriends OR interpersonal OR “gender based” OR gender-based) |  |
| 4 | DE "Violence" OR DE "Domestic Violence" OR DE "Gender Violence" OR DE "Gun Violence" OR DE "Sexual Violence" OR DE "Violent Crime" OR DE "Virtual Violence" OR DE "Relational Aggression" OR DE "Bullying" OR DE "Cyberbullying" OR DE "Harassment" OR DE "Sexual Harassment" OR DE "Stalking" OR DE "Workplace Harassment" OR DE "Emotional Abuse" OR TI (violence OR cyberviolence OR violent OR abuse OR abused OR abuses OR abuser OR abusers OR cyberabuse OR cyberabuses OR cyberabuser OR cyberabusers OR assault OR assaulted OR assaults OR assaulter OR assaulters OR cyberassault OR cyberassaulted OR cyberassaults OR cyberassaulter OR cyberassaulters OR assailant OR assailants OR battery OR battered OR batterer OR batterers OR beat OR beaten OR beats OR beating OR perpetrator OR perpetrators OR perpetration OR perpetrating OR perpetrated OR perpetrate OR perpetrates OR rape OR raped OR rapes OR rapist OR rapists OR mistreatment OR mistreated OR mistreatments OR molestation OR molested OR molestations OR molester OR molesters OR attacked OR attacks OR attack OR attacker OR attackers OR cyberattacked OR cyberattacks OR cyberattack OR cyberattacker OR cyberattackers OR coercive OR coercion OR aggression OR aggressive OR aggressor OR aggressors OR cyberaggression OR harassment OR harassments OR harassed OR harass OR harasses OR harassing OR stalking OR stalked OR stalker OR stalkers OR cyberstalking OR cyberstalked OR cyberstalker OR cyberstalkers OR victimization OR victimized OR victimize OR victimizes OR victimizing OR cybervictimization OR cybervictimized OR cybervictimize OR cybervictimizes OR cybervictimizing OR terrorism OR cyberterrorism OR bullying OR bullied OR bully OR bullies OR cyberbullying OR cyberbullied OR cyberbully OR cyberbullies OR blackmail OR blackmailed OR blackmails OR blackmailing OR bribery OR bribe OR bribes OR bribed OR bribing OR incivility OR cyberincivility OR defamation OR defames OR defamed OR defaming OR slander OR slanderous OR exploitation OR exploited OR exploit OR exploits OR exploiting OR sexploitation OR “hate speech” OR sextortion OR “sexual solicitation” OR “unwanted sex” OR “unwanted sexual” OR extortion OR doxxing OR hacking OR “online impersonation” OR “false identity” OR “false identities” OR “non-consensual sharing” OR shallowfake OR shallowfakes OR deepfake OR deepfakes OR “predatory behavior” OR “predatory behaviors” OR predator OR predators OR predatorial OR cyberpredator OR cyberpredators) OR AB (violence OR cyberviolence OR violent OR abuse OR abused OR abuses OR abuser OR abusers OR cyberabuse OR cyberabuses OR cyberabuser OR cyberabusers OR assault OR assaulted OR assaults OR assaulter OR assaulters OR cyberassault OR cyberassaulted OR cyberassaults OR cyberassaulter OR cyberassaulters OR assailant OR assailants OR battery OR battered OR batterer OR batterers OR beat OR beaten OR beats OR beating OR perpetrator OR perpetrators OR perpetration OR perpetrating OR perpetrated OR perpetrate OR perpetrates OR rape OR raped OR rapes OR rapist OR rapists OR mistreatment OR mistreated OR mistreatments OR molestation OR molested OR molestations OR molester OR molesters OR attacked OR attacks OR attack OR attacker OR attackers OR cyberattacked OR cyberattacks OR cyberattack OR cyberattacker OR cyberattackers OR coercive OR coercion OR aggression OR aggressive OR aggressor OR aggressors OR cyberaggression OR harassment OR harassments OR harassed OR harass OR harasses OR harassing OR stalking OR stalked OR stalker OR stalkers OR cyberstalking OR cyberstalked OR cyberstalker OR cyberstalkers OR victimization OR victimized OR victimize OR victimizes OR victimizing OR cybervictimization OR cybervictimized OR cybervictimize OR cybervictimizes OR cybervictimizing OR terrorism OR cyberterrorism OR bullying OR bullied OR bully OR bullies OR cyberbullying OR cyberbullied OR cyberbully OR cyberbullies OR blackmail OR blackmailed OR blackmails OR blackmailing OR bribery OR bribe OR bribes OR bribed OR bribing OR incivility OR cyberincivility OR defamation OR defames OR defamed OR defaming OR slander OR slanderous OR exploitation OR exploited OR exploit OR exploits OR exploiting OR sexploitation OR “hate speech” OR sextortion OR “sexual solicitation” OR “unwanted sex” OR “unwanted sexual” OR extortion OR doxxing OR hacking OR “online impersonation” OR “false identity” OR “false identities” OR “non-consensual sharing” OR shallowfake OR shallowfakes OR deepfake OR deepfakes OR “predatory behavior” OR “predatory behaviors” OR predator OR predators OR predatorial OR cyberpredator OR cyberpredators) |  |
| 5 | #1 AND #2 AND #3 AND #4 | 754 |
| 6 | #5 AND **Source Types:** Academic Journals | 488 |

Database: CINAHL Plus with Full Text (EBSCO*host*)

| Set # |  | Results |
| --- | --- | --- |
| 1 | MH "Hispanic Americans+" OR TI (Hispanic OR Hispanics OR Latina OR Latinas OR Latino OR Latinos OR Latine OR Latinx OR Chicana OR Chicanas OR Chicano OR Chicanos OR “Cuban American” OR “Cuban Americans” OR “Mexican American” OR “Mexican Americans” OR “Puerto Rican” OR “Puerto Ricans” OR “Spanish American” OR “Spanish Americans” OR “US Latin American” OR “US Latin Americans”) OR AB (Hispanic OR Hispanics OR Latina OR Latinas OR Latino OR Latinos OR Latine OR Latinx OR Chicana OR Chicanas OR Chicano OR Chicanos OR “Cuban American” OR “Cuban Americans” OR “Mexican American” OR “Mexican Americans” OR “Puerto Rican” OR “Puerto Ricans” OR “Spanish American” OR “Spanish Americans” OR “US Latin American” OR “US Latin Americans”) |  |
| 2 | MH "Immigrants+" OR MH "Emigration and Immigration+" OR MH "Migrants" OR MH "Refugees" OR MH "Acculturation" OR TI (migrant OR migrants OR immigrant OR immigrants OR immigration OR migration OR immigrated OR migrated OR immigrating OR migrating OR immigrate OR migrate OR immigrates OR migrates OR refugee OR refugees OR asylum OR displaced OR acculturation OR acculturate OR acculturates OR acculturated OR acculturating OR assimilation OR assimilate OR assimilates OR assimilated OR assimilating) OR AB (migrant OR migrants OR immigrant OR immigrants OR immigration OR migration OR immigrated OR migrated OR immigrating OR migrating OR immigrate OR migrate OR immigrates OR migrates OR refugee OR refugees OR asylum OR displaced OR acculturation OR acculturate OR acculturates OR acculturated OR acculturating OR assimilation OR assimilate OR assimilates OR assimilated OR assimilating) |  |
| 3 | MH "Family+" OR MH "Marriage+" OR MH "Sexual Partners" OR MH "Abused Women" OR TI (domestic OR family OR families OR partner OR partners OR spouse OR spouses OR spousal OR marriage OR married OR marital OR couple OR couples OR husband OR husbands OR wife OR wives OR dating OR date OR boyfriend OR boyfriends OR girlfriend OR girlfriends OR interpersonal OR “gender based” OR gender-based) OR AB (domestic OR family OR families OR partner OR partners OR spouse OR spouses OR spousal OR marriage OR married OR marital OR couple OR couples OR husband OR husbands OR wife OR wives OR dating OR date OR boyfriend OR boyfriends OR girlfriend OR girlfriends OR interpersonal OR “gender based” OR gender-based) |  |
| 4 | MH "Violence+" OR MH "Aggression+" OR MH "Sexual Harassment" OR MH "Incivility" OR TI (violence OR cyberviolence OR violent OR abuse OR abused OR abuses OR abuser OR abusers OR cyberabuse OR cyberabuses OR cyberabuser OR cyberabusers OR assault OR assaulted OR assaults OR assaulter OR assaulters OR cyberassault OR cyberassaulted OR cyberassaults OR cyberassaulter OR cyberassaulters OR assailant OR assailants OR battery OR battered OR batterer OR batterers OR beat OR beaten OR beats OR beating OR perpetrator OR perpetrators OR perpetration OR perpetrating OR perpetrated OR perpetrate OR perpetrates OR rape OR raped OR rapes OR rapist OR rapists OR mistreatment OR mistreated OR mistreatments OR molestation OR molested OR molestations OR molester OR molesters OR attacked OR attacks OR attack OR attacker OR attackers OR cyberattacked OR cyberattacks OR cyberattack OR cyberattacker OR cyberattackers OR coercive OR coercion OR aggression OR aggressive OR aggressor OR aggressors OR cyberaggression OR harassment OR harassments OR harassed OR harass OR harasses OR harassing OR stalking OR stalked OR stalker OR stalkers OR cyberstalking OR cyberstalked OR cyberstalker OR cyberstalkers OR victimization OR victimized OR victimize OR victimizes OR victimizing OR cybervictimization OR cybervictimized OR cybervictimize OR cybervictimizes OR cybervictimizing OR terrorism OR cyberterrorism OR bullying OR bullied OR bully OR bullies OR cyberbullying OR cyberbullied OR cyberbully OR cyberbullies OR blackmail OR blackmailed OR blackmails OR blackmailing OR bribery OR bribe OR bribes OR bribed OR bribing OR incivility OR cyberincivility OR defamation OR defames OR defamed OR defaming OR slander OR slanderous OR exploitation OR exploited OR exploit OR exploits OR exploiting OR sexploitation OR “hate speech” OR sextortion OR “sexual solicitation” OR “unwanted sex” OR “unwanted sexual” OR extortion OR doxxing OR hacking OR “online impersonation” OR “false identity” OR “false identities” OR “non-consensual sharing” OR shallowfake OR shallowfakes OR deepfake OR deepfakes OR “predatory behavior” OR “predatory behaviors” OR predator OR predators OR predatorial OR cyberpredator OR cyberpredators) OR AB (violence OR cyberviolence OR violent OR abuse OR abused OR abuses OR abuser OR abusers OR cyberabuse OR cyberabuses OR cyberabuser OR cyberabusers OR assault OR assaulted OR assaults OR assaulter OR assaulters OR cyberassault OR cyberassaulted OR cyberassaults OR cyberassaulter OR cyberassaulters OR assailant OR assailants OR battery OR battered OR batterer OR batterers OR beat OR beaten OR beats OR beating OR perpetrator OR perpetrators OR perpetration OR perpetrating OR perpetrated OR perpetrate OR perpetrates OR rape OR raped OR rapes OR rapist OR rapists OR mistreatment OR mistreated OR mistreatments OR molestation OR molested OR molestations OR molester OR molesters OR attacked OR attacks OR attack OR attacker OR attackers OR cyberattacked OR cyberattacks OR cyberattack OR cyberattacker OR cyberattackers OR coercive OR coercion OR aggression OR aggressive OR aggressor OR aggressors OR cyberaggression OR harassment OR harassments OR harassed OR harass OR harasses OR harassing OR stalking OR stalked OR stalker OR stalkers OR cyberstalking OR cyberstalked OR cyberstalker OR cyberstalkers OR victimization OR victimized OR victimize OR victimizes OR victimizing OR cybervictimization OR cybervictimized OR cybervictimize OR cybervictimizes OR cybervictimizing OR terrorism OR cyberterrorism OR bullying OR bullied OR bully OR bullies OR cyberbullying OR cyberbullied OR cyberbully OR cyberbullies OR blackmail OR blackmailed OR blackmails OR blackmailing OR bribery OR bribe OR bribes OR bribed OR bribing OR incivility OR cyberincivility OR defamation OR defames OR defamed OR defaming OR slander OR slanderous OR exploitation OR exploited OR exploit OR exploits OR exploiting OR sexploitation OR “hate speech” OR sextortion OR “sexual solicitation” OR “unwanted sex” OR “unwanted sexual” OR extortion OR doxxing OR hacking OR “online impersonation” OR “false identity” OR “false identities” OR “non-consensual sharing” OR shallowfake OR shallowfakes OR deepfake OR deepfakes OR “predatory behavior” OR “predatory behaviors” OR predator OR predators OR predatorial OR cyberpredator OR cyberpredators) |  |
| 5 | #1 AND #2 AND #3 AND #4 | 363 |
| 6 | #5 AND **Source Types:** Academic Journals | 347 |

Database: Scopus

| Set # |  | Results |
| --- | --- | --- |
| 1 | TITLE-ABS (Hispanic OR Hispanics OR Latina OR Latinas OR Latino OR Latinos OR Latine OR Latinx OR Chicana OR Chicanas OR Chicano OR Chicanos OR “Cuban American” OR “Cuban Americans” OR “Mexican American” OR “Mexican Americans” OR “Puerto Rican” OR “Puerto Ricans” OR “Spanish American” OR “Spanish Americans” OR “US Latin American” OR “US Latin Americans”) |  |
| 2 | TITLE-ABS (migrant OR migrants OR immigrant OR immigrants OR immigration OR migration OR immigrated OR migrated OR immigrating OR migrating OR immigrate OR migrate OR immigrates OR migrates OR refugee OR refugees OR asylum OR displaced OR acculturation OR acculturate OR acculturates OR acculturated OR acculturating OR assimilation OR assimilate OR assimilates OR assimilated OR assimilating) |  |
| 3 | TITLE-ABS (domestic OR family OR families OR partner OR partners OR spouse OR spouses OR spousal OR marriage OR married OR marital OR couple OR couples OR husband OR husbands OR wife OR wives OR dating OR date OR boyfriend OR boyfriends OR girlfriend OR girlfriends OR interpersonal OR “gender based” OR gender-based) |  |
| 4 | TITLE-ABS (violence OR cyberviolence OR violent OR abuse OR abused OR abuses OR abuser OR abusers OR cyberabuse OR cyberabuses OR cyberabuser OR cyberabusers OR assault OR assaulted OR assaults OR assaulter OR assaulters OR cyberassault OR cyberassaulted OR cyberassaults OR cyberassaulter OR cyberassaulters OR assailant OR assailants OR battery OR battered OR batterer OR batterers OR beat OR beaten OR beats OR beating OR perpetrator OR perpetrators OR perpetration OR perpetrating OR perpetrated OR perpetrate OR perpetrates OR rape OR raped OR rapes OR rapist OR rapists OR mistreatment OR mistreated OR mistreatments OR molestation OR molested OR molestations OR molester OR molesters OR attacked OR attacks OR attack OR attacker OR attackers OR cyberattacked OR cyberattacks OR cyberattack OR cyberattacker OR cyberattackers OR coercive OR coercion OR aggression OR aggressive OR aggressor OR aggressors OR cyberaggression OR harassment OR harassments OR harassed OR harass OR harasses OR harassing OR stalking OR stalked OR stalker OR stalkers OR cyberstalking OR cyberstalked OR cyberstalker OR cyberstalkers OR victimization OR victimized OR victimize OR victimizes OR victimizing OR cybervictimization OR cybervictimized OR cybervictimize OR cybervictimizes OR cybervictimizing OR terrorism OR cyberterrorism OR bullying OR bullied OR bully OR bullies OR cyberbullying OR cyberbullied OR cyberbully OR cyberbullies OR blackmail OR blackmailed OR blackmails OR blackmailing OR bribery OR bribe OR bribes OR bribed OR bribing OR incivility OR cyberincivility OR defamation OR defames OR defamed OR defaming OR slander OR slanderous OR exploitation OR exploited OR exploit OR exploits OR exploiting OR sexploitation OR “hate speech” OR sextortion OR “sexual solicitation” OR “unwanted sex” OR “unwanted sexual” OR extortion OR doxxing OR hacking OR “online impersonation” OR “false identity” OR “false identities” OR “non-consensual sharing” OR shallowfake OR shallowfakes OR deepfake OR deepfakes OR “predatory behavior” OR “predatory behaviors” OR predator OR predators OR predatorial OR cyberpredator OR cyberpredators) |  |
| 5 | #1 AND #2 AND #3 AND #4 | 616 |
| 6 | #5 AND ( LIMIT-TO ( DOCTYPE , "ar" ) OR LIMIT-TO ( DOCTYPE , "re" ) OR LIMIT-TO ( DOCTYPE , "er" ) ) | 517 |

Database: Social Work Abstracts (EBSCO*host*)

| Set # |  | Results |
| --- | --- | --- |
| 1 | ZU "hispanics" OR TI (Hispanic OR Hispanics OR Latina OR Latinas OR Latino OR Latinos OR Latine OR Latinx OR Chicana OR Chicanas OR Chicano OR Chicanos OR “Cuban American” OR “Cuban Americans” OR “Mexican American” OR “Mexican Americans” OR “Puerto Rican” OR “Puerto Ricans” OR “Spanish American” OR “Spanish Americans” OR “US Latin American” OR “US Latin Americans”) OR AB (Hispanic OR Hispanics OR Latina OR Latinas OR Latino OR Latinos OR Latine OR Latinx OR Chicana OR Chicanas OR Chicano OR Chicanos OR “Cuban American” OR “Cuban Americans” OR “Mexican American” OR “Mexican Americans” OR “Puerto Rican” OR “Puerto Ricans” OR “Spanish American” OR “Spanish Americans” OR “US Latin American” OR “US Latin Americans”) |  |
| 2 | ZU "immigrants" OR ZU "immigration" OR ZU "migrants" OR ZU "refugees" OR ZU "acculturation" OR TI (migrant OR migrants OR immigrant OR immigrants OR immigration OR migration OR immigrated OR migrated OR immigrating OR migrating OR immigrate OR migrate OR immigrates OR migrates OR refugee OR refugees OR asylum OR displaced OR acculturation OR acculturate OR acculturates OR acculturated OR acculturating OR assimilation OR assimilate OR assimilates OR assimilated OR assimilating) OR AB (migrant OR migrants OR immigrant OR immigrants OR immigration OR migration OR immigrated OR migrated OR immigrating OR migrating OR immigrate OR migrate OR immigrates OR migrates OR refugee OR refugees OR asylum OR displaced OR acculturation OR acculturate OR acculturates OR acculturated OR acculturating OR assimilation OR assimilate OR assimilates OR assimilated OR assimilating) |  |
| 3 | ZU "family" OR ZU "marriage" OR ZU "battered women" OR TI (domestic OR family OR families OR partner OR partners OR spouse OR spouses OR spousal OR marriage OR married OR marital OR couple OR couples OR husband OR husbands OR wife OR wives OR dating OR date OR boyfriend OR boyfriends OR girlfriend OR girlfriends OR interpersonal OR “gender based” OR gender-based) OR AB (domestic OR family OR families OR partner OR partners OR spouse OR spouses OR spousal OR marriage OR married OR marital OR couple OR couples OR husband OR husbands OR wife OR wives OR dating OR date OR boyfriend OR boyfriends OR girlfriend OR girlfriends OR interpersonal OR “gender based” OR gender-based) |  |
| 4 | ZU "violence" OR ZU "intimate partner violence" OR ZU "domestic violence" OR ZU "aggression" OR ZU "bullying" OR ZU "emotional abuse" OR TI (violence OR cyberviolence OR violent OR abuse OR abused OR abuses OR abuser OR abusers OR cyberabuse OR cyberabuses OR cyberabuser OR cyberabusers OR assault OR assaulted OR assaults OR assaulter OR assaulters OR cyberassault OR cyberassaulted OR cyberassaults OR cyberassaulter OR cyberassaulters OR assailant OR assailants OR battery OR battered OR batterer OR batterers OR beat OR beaten OR beats OR beating OR perpetrator OR perpetrators OR perpetration OR perpetrating OR perpetrated OR perpetrate OR perpetrates OR rape OR raped OR rapes OR rapist OR rapists OR mistreatment OR mistreated OR mistreatments OR molestation OR molested OR molestations OR molester OR molesters OR attacked OR attacks OR attack OR attacker OR attackers OR cyberattacked OR cyberattacks OR cyberattack OR cyberattacker OR cyberattackers OR coercive OR coercion OR aggression OR aggressive OR aggressor OR aggressors OR cyberaggression OR harassment OR harassments OR harassed OR harass OR harasses OR harassing OR stalking OR stalked OR stalker OR stalkers OR cyberstalking OR cyberstalked OR cyberstalker OR cyberstalkers OR victimization OR victimized OR victimize OR victimizes OR victimizing OR cybervictimization OR cybervictimized OR cybervictimize OR cybervictimizes OR cybervictimizing OR terrorism OR cyberterrorism OR bullying OR bullied OR bully OR bullies OR cyberbullying OR cyberbullied OR cyberbully OR cyberbullies OR blackmail OR blackmailed OR blackmails OR blackmailing OR bribery OR bribe OR bribes OR bribed OR bribing OR incivility OR cyberincivility OR defamation OR defames OR defamed OR defaming OR slander OR slanderous OR exploitation OR exploited OR exploit OR exploits OR exploiting OR sexploitation OR “hate speech” OR sextortion OR “sexual solicitation” OR “unwanted sex” OR “unwanted sexual” OR extortion OR doxxing OR hacking OR “online impersonation” OR “false identity” OR “false identities” OR “non-consensual sharing” OR shallowfake OR shallowfakes OR deepfake OR deepfakes OR “predatory behavior” OR “predatory behaviors” OR predator OR predators OR predatorial OR cyberpredator OR cyberpredators) OR AB (violence OR cyberviolence OR violent OR abuse OR abused OR abuses OR abuser OR abusers OR cyberabuse OR cyberabuses OR cyberabuser OR cyberabusers OR assault OR assaulted OR assaults OR assaulter OR assaulters OR cyberassault OR cyberassaulted OR cyberassaults OR cyberassaulter OR cyberassaulters OR assailant OR assailants OR battery OR battered OR batterer OR batterers OR beat OR beaten OR beats OR beating OR perpetrator OR perpetrators OR perpetration OR perpetrating OR perpetrated OR perpetrate OR perpetrates OR rape OR raped OR rapes OR rapist OR rapists OR mistreatment OR mistreated OR mistreatments OR molestation OR molested OR molestations OR molester OR molesters OR attacked OR attacks OR attack OR attacker OR attackers OR cyberattacked OR cyberattacks OR cyberattack OR cyberattacker OR cyberattackers OR coercive OR coercion OR aggression OR aggressive OR aggressor OR aggressors OR cyberaggression OR harassment OR harassments OR harassed OR harass OR harasses OR harassing OR stalking OR stalked OR stalker OR stalkers OR cyberstalking OR cyberstalked OR cyberstalker OR cyberstalkers OR victimization OR victimized OR victimize OR victimizes OR victimizing OR cybervictimization OR cybervictimized OR cybervictimize OR cybervictimizes OR cybervictimizing OR terrorism OR cyberterrorism OR bullying OR bullied OR bully OR bullies OR cyberbullying OR cyberbullied OR cyberbully OR cyberbullies OR blackmail OR blackmailed OR blackmails OR blackmailing OR bribery OR bribe OR bribes OR bribed OR bribing OR incivility OR cyberincivility OR defamation OR defames OR defamed OR defaming OR slander OR slanderous OR exploitation OR exploited OR exploit OR exploits OR exploiting OR sexploitation OR “hate speech” OR sextortion OR “sexual solicitation” OR “unwanted sex” OR “unwanted sexual” OR extortion OR doxxing OR hacking OR “online impersonation” OR “false identity” OR “false identities” OR “non-consensual sharing” OR shallowfake OR shallowfakes OR deepfake OR deepfakes OR “predatory behavior” OR “predatory behaviors” OR predator OR predators OR predatorial OR cyberpredator OR cyberpredators) |  |
| 5 | #1 AND #2 AND #3 AND #4 | 27 |
| 6 | #5 AND **Source Types:** All Journals | 25 |

Database: Sociological Abstracts (ProQuest)

| Set # |  | Results |
| --- | --- | --- |
| 1 | MAINSUBJECT.EXACT.EXPLODE("Hispanic Americans") OR title(Hispanic OR Hispanics OR Latina OR Latinas OR Latino OR Latinos OR Latine OR Latinx OR Chicana OR Chicanas OR Chicano OR Chicanos OR “Cuban American” OR “Cuban Americans” OR “Mexican American” OR “Mexican Americans” OR “Puerto Rican” OR “Puerto Ricans” OR “Spanish American” OR “Spanish Americans” OR “US Latin American” OR “US Latin Americans”) OR abstract(Hispanic OR Hispanics OR Latina OR Latinas OR Latino OR Latinos OR Latine OR Latinx OR Chicana OR Chicanas OR Chicano OR Chicanos OR “Cuban American” OR “Cuban Americans” OR “Mexican American” OR “Mexican Americans” OR “Puerto Rican” OR “Puerto Ricans” OR “Spanish American” OR “Spanish Americans” OR “US Latin American” OR “US Latin Americans”) |  |
| 2 | MAINSUBJECT.EXACT.EXPLODE("Immigrants") OR MAINSUBJECT.EXACT.EXPLODE("Immigration") OR MAINSUBJECT.EXACT.EXPLODE("Migrants") OR MAINSUBJECT.EXACT.EXPLODE("Refugees") OR MAINSUBJECT.EXACT.EXPLODE("Acculturation") OR title(migrant OR migrants OR immigrant OR immigrants OR immigration OR migration OR immigrated OR migrated OR immigrating OR migrating OR immigrate OR migrate OR immigrates OR migrates OR refugee OR refugees OR asylum OR displaced OR acculturation OR acculturate OR acculturates OR acculturated OR acculturating OR assimilation OR assimilate OR assimilates OR assimilated OR assimilating) OR abstract(migrant OR migrants OR immigrant OR immigrants OR immigration OR migration OR immigrated OR migrated OR immigrating OR migrating OR immigrate OR migrate OR immigrates OR migrates OR refugee OR refugees OR asylum OR displaced OR acculturation OR acculturate OR acculturates OR acculturated OR acculturating OR assimilation OR assimilate OR assimilates OR assimilated OR assimilating) |  |
| 3 | MAINSUBJECT.EXACT.EXPLODE("Families & family life") OR MAINSUBJECT.EXACT.EXPLODE("Marriage") OR MAINSUBJECT.EXACT.EXPLODE("Abused women") OR title(domestic OR family OR families OR partner OR partners OR spouse OR spouses OR spousal OR marriage OR married OR marital OR couple OR couples OR husband OR husbands OR wife OR wives OR dating OR date OR boyfriend OR boyfriends OR girlfriend OR girlfriends OR interpersonal OR “gender based” OR gender-based) OR abstract(domestic OR family OR families OR partner OR partners OR spouse OR spouses OR spousal OR marriage OR married OR marital OR couple OR couples OR husband OR husbands OR wife OR wives OR dating OR date OR boyfriend OR boyfriends OR girlfriend OR girlfriends OR interpersonal OR “gender based” OR gender-based) |  |
| 4 | MAINSUBJECT.EXACT.EXPLODE("Intimate partner violence") OR MAINSUBJECT.EXACT.EXPLODE("Domestic violence") OR MAINSUBJECT.EXACT.EXPLODE("Gender-based violence") OR MAINSUBJECT.EXACT.EXPLODE("Aggressiveness") OR MAINSUBJECT.EXACT.EXPLODE("Emotional abuse") OR title(violence OR cyberviolence OR violent OR abuse OR abused OR abuses OR abuser OR abusers OR cyberabuse OR cyberabuses OR cyberabuser OR cyberabusers OR assault OR assaulted OR assaults OR assaulter OR assaulters OR cyberassault OR cyberassaulted OR cyberassaults OR cyberassaulter OR cyberassaulters OR assailant OR assailants OR battery OR battered OR batterer OR batterers OR beat OR beaten OR beats OR beating OR perpetrator OR perpetrators OR perpetration OR perpetrating OR perpetrated OR perpetrate OR perpetrates OR rape OR raped OR rapes OR rapist OR rapists OR mistreatment OR mistreated OR mistreatments OR molestation OR molested OR molestations OR molester OR molesters OR attacked OR attacks OR attack OR attacker OR attackers OR cyberattacked OR cyberattacks OR cyberattack OR cyberattacker OR cyberattackers OR coercive OR coercion OR aggression OR aggressive OR aggressor OR aggressors OR cyberaggression OR harassment OR harassments OR harassed OR harass OR harasses OR harassing OR stalking OR stalked OR stalker OR stalkers OR cyberstalking OR cyberstalked OR cyberstalker OR cyberstalkers OR victimization OR victimized OR victimize OR victimizes OR victimizing OR cybervictimization OR cybervictimized OR cybervictimize OR cybervictimizes OR cybervictimizing OR terrorism OR cyberterrorism OR bullying OR bullied OR bully OR bullies OR cyberbullying OR cyberbullied OR cyberbully OR cyberbullies OR blackmail OR blackmailed OR blackmails OR blackmailing OR bribery OR bribe OR bribes OR bribed OR bribing OR incivility OR cyberincivility OR defamation OR defames OR defamed OR defaming OR slander OR slanderous OR exploitation OR exploited OR exploit OR exploits OR exploiting OR sexploitation OR “hate speech” OR sextortion OR “sexual solicitation” OR “unwanted sex” OR “unwanted sexual” OR extortion OR doxxing OR hacking OR “online impersonation” OR “false identity” OR “false identities” OR “non-consensual sharing” OR shallowfake OR shallowfakes OR deepfake OR deepfakes OR “predatory behavior” OR “predatory behaviors” OR predator OR predators OR predatorial OR cyberpredator OR cyberpredators) OR abstract(violence OR cyberviolence OR violent OR abuse OR abused OR abuses OR abuser OR abusers OR cyberabuse OR cyberabuses OR cyberabuser OR cyberabusers OR assault OR assaulted OR assaults OR assaulter OR assaulters OR cyberassault OR cyberassaulted OR cyberassaults OR cyberassaulter OR cyberassaulters OR assailant OR assailants OR battery OR battered OR batterer OR batterers OR beat OR beaten OR beats OR beating OR perpetrator OR perpetrators OR perpetration OR perpetrating OR perpetrated OR perpetrate OR perpetrates OR rape OR raped OR rapes OR rapist OR rapists OR mistreatment OR mistreated OR mistreatments OR molestation OR molested OR molestations OR molester OR molesters OR attacked OR attacks OR attack OR attacker OR attackers OR cyberattacked OR cyberattacks OR cyberattack OR cyberattacker OR cyberattackers OR coercive OR coercion OR aggression OR aggressive OR aggressor OR aggressors OR cyberaggression OR harassment OR harassments OR harassed OR harass OR harasses OR harassing OR stalking OR stalked OR stalker OR stalkers OR cyberstalking OR cyberstalked OR cyberstalker OR cyberstalkers OR victimization OR victimized OR victimize OR victimizes OR victimizing OR cybervictimization OR cybervictimized OR cybervictimize OR cybervictimizes OR cybervictimizing OR terrorism OR cyberterrorism OR bullying OR bullied OR bully OR bullies OR cyberbullying OR cyberbullied OR cyberbully OR cyberbullies OR blackmail OR blackmailed OR blackmails OR blackmailing OR bribery OR bribe OR bribes OR bribed OR bribing OR incivility OR cyberincivility OR defamation OR defames OR defamed OR defaming OR slander OR slanderous OR exploitation OR exploited OR exploit OR exploits OR exploiting OR sexploitation OR “hate speech” OR sextortion OR “sexual solicitation” OR “unwanted sex” OR “unwanted sexual” OR extortion OR doxxing OR hacking OR “online impersonation” OR “false identity” OR “false identities” OR “non-consensual sharing” OR shallowfake OR shallowfakes OR deepfake OR deepfakes OR “predatory behavior” OR “predatory behaviors” OR predator OR predators OR predatorial OR cyberpredator OR cyberpredators) |  |
| 5 | #1 AND #2 AND #3 AND #4 | 518 |
| 6 | #5 AND **Applied filters: Scholarly Journals** | 419 |

Database: GenderWatch Collection (ProQuest)

| Set # |  | Results |
| --- | --- | --- |
| 1 | MAINSUBJECT.EXACT("Hispanic Americans") OR title(Hispanic OR Hispanics OR Latina OR Latinas OR Latino OR Latinos OR Latine OR Latinx OR Chicana OR Chicanas OR Chicano OR Chicanos OR “Cuban American” OR “Cuban Americans” OR “Mexican American” OR “Mexican Americans” OR “Puerto Rican” OR “Puerto Ricans” OR “Spanish American” OR “Spanish Americans” OR “US Latin American” OR “US Latin Americans”) OR abstract(Hispanic OR Hispanics OR Latina OR Latinas OR Latino OR Latinos OR Latine OR Latinx OR Chicana OR Chicanas OR Chicano OR Chicanos OR “Cuban American” OR “Cuban Americans” OR “Mexican American” OR “Mexican Americans” OR “Puerto Rican” OR “Puerto Ricans” OR “Spanish American” OR “Spanish Americans” OR “US Latin American” OR “US Latin Americans”) |  |
| 2 | MAINSUBJECT.EXACT("Immigrants") OR MAINSUBJECT.EXACT("Immigration") OR MAINSUBJECT.EXACT("Migrants") OR MAINSUBJECT.EXACT("Refugees") OR MAINSUBJECT.EXACT("Acculturation") OR title(migrant OR migrants OR immigrant OR immigrants OR immigration OR migration OR immigrated OR migrated OR immigrating OR migrating OR immigrate OR migrate OR immigrates OR migrates OR refugee OR refugees OR asylum OR displaced OR acculturation OR acculturate OR acculturates OR acculturated OR acculturating OR assimilation OR assimilate OR assimilates OR assimilated OR assimilating) OR abstract(migrant OR migrants OR immigrant OR immigrants OR immigration OR migration OR immigrated OR migrated OR immigrating OR migrating OR immigrate OR migrate OR immigrates OR migrates OR refugee OR refugees OR asylum OR displaced OR acculturation OR acculturate OR acculturates OR acculturated OR acculturating OR assimilation OR assimilate OR assimilates OR assimilated OR assimilating) |  |
| 3 | MAINSUBJECT.EXACT("Families & family life") OR MAINSUBJECT.EXACT("Marriage") OR MAINSUBJECT.EXACT("Abused women") OR title(domestic OR family OR families OR partner OR partners OR spouse OR spouses OR spousal OR marriage OR married OR marital OR couple OR couples OR husband OR husbands OR wife OR wives OR dating OR date OR boyfriend OR boyfriends OR girlfriend OR girlfriends OR interpersonal OR “gender based” OR gender-based) OR abstract(domestic OR family OR families OR partner OR partners OR spouse OR spouses OR spousal OR marriage OR married OR marital OR couple OR couples OR husband OR husbands OR wife OR wives OR dating OR date OR boyfriend OR boyfriends OR girlfriend OR girlfriends OR interpersonal OR “gender based” OR gender-based) |  |
| 4 | MAINSUBJECT.EXACT("Intimate partner violence") OR MAINSUBJECT.EXACT("Domestic violence") OR MAINSUBJECT.EXACT("Gender-based violence") OR MAINSUBJECT.EXACT("Aggressiveness") OR MAINSUBJECT.EXACT("Emotional abuse") OR title(violence OR cyberviolence OR violent OR abuse OR abused OR abuses OR abuser OR abusers OR cyberabuse OR cyberabuses OR cyberabuser OR cyberabusers OR assault OR assaulted OR assaults OR assaulter OR assaulters OR cyberassault OR cyberassaulted OR cyberassaults OR cyberassaulter OR cyberassaulters OR assailant OR assailants OR battery OR battered OR batterer OR batterers OR beat OR beaten OR beats OR beating OR perpetrator OR perpetrators OR perpetration OR perpetrating OR perpetrated OR perpetrate OR perpetrates OR rape OR raped OR rapes OR rapist OR rapists OR mistreatment OR mistreated OR mistreatments OR molestation OR molested OR molestations OR molester OR molesters OR attacked OR attacks OR attack OR attacker OR attackers OR cyberattacked OR cyberattacks OR cyberattack OR cyberattacker OR cyberattackers OR coercive OR coercion OR aggression OR aggressive OR aggressor OR aggressors OR cyberaggression OR harassment OR harassments OR harassed OR harass OR harasses OR harassing OR stalking OR stalked OR stalker OR stalkers OR cyberstalking OR cyberstalked OR cyberstalker OR cyberstalkers OR victimization OR victimized OR victimize OR victimizes OR victimizing OR cybervictimization OR cybervictimized OR cybervictimize OR cybervictimizes OR cybervictimizing OR terrorism OR cyberterrorism OR bullying OR bullied OR bully OR bullies OR cyberbullying OR cyberbullied OR cyberbully OR cyberbullies OR blackmail OR blackmailed OR blackmails OR blackmailing OR bribery OR bribe OR bribes OR bribed OR bribing OR incivility OR cyberincivility OR defamation OR defames OR defamed OR defaming OR slander OR slanderous OR exploitation OR exploited OR exploit OR exploits OR exploiting OR sexploitation OR “hate speech” OR sextortion OR “sexual solicitation” OR “unwanted sex” OR “unwanted sexual” OR extortion OR doxxing OR hacking OR “online impersonation” OR “false identity” OR “false identities” OR “non-consensual sharing” OR shallowfake OR shallowfakes OR deepfake OR deepfakes OR “predatory behavior” OR “predatory behaviors” OR predator OR predators OR predatorial OR cyberpredator OR cyberpredators) OR abstract(violence OR cyberviolence OR violent OR abuse OR abused OR abuses OR abuser OR abusers OR cyberabuse OR cyberabuses OR cyberabuser OR cyberabusers OR assault OR assaulted OR assaults OR assaulter OR assaulters OR cyberassault OR cyberassaulted OR cyberassaults OR cyberassaulter OR cyberassaulters OR assailant OR assailants OR battery OR battered OR batterer OR batterers OR beat OR beaten OR beats OR beating OR perpetrator OR perpetrators OR perpetration OR perpetrating OR perpetrated OR perpetrate OR perpetrates OR rape OR raped OR rapes OR rapist OR rapists OR mistreatment OR mistreated OR mistreatments OR molestation OR molested OR molestations OR molester OR molesters OR attacked OR attacks OR attack OR attacker OR attackers OR cyberattacked OR cyberattacks OR cyberattack OR cyberattacker OR cyberattackers OR coercive OR coercion OR aggression OR aggressive OR aggressor OR aggressors OR cyberaggression OR harassment OR harassments OR harassed OR harass OR harasses OR harassing OR stalking OR stalked OR stalker OR stalkers OR cyberstalking OR cyberstalked OR cyberstalker OR cyberstalkers OR victimization OR victimized OR victimize OR victimizes OR victimizing OR cybervictimization OR cybervictimized OR cybervictimize OR cybervictimizes OR cybervictimizing OR terrorism OR cyberterrorism OR bullying OR bullied OR bully OR bullies OR cyberbullying OR cyberbullied OR cyberbully OR cyberbullies OR blackmail OR blackmailed OR blackmails OR blackmailing OR bribery OR bribe OR bribes OR bribed OR bribing OR incivility OR cyberincivility OR defamation OR defames OR defamed OR defaming OR slander OR slanderous OR exploitation OR exploited OR exploit OR exploits OR exploiting OR sexploitation OR “hate speech” OR sextortion OR “sexual solicitation” OR “unwanted sex” OR “unwanted sexual” OR extortion OR doxxing OR hacking OR “online impersonation” OR “false identity” OR “false identities” OR “non-consensual sharing” OR shallowfake OR shallowfakes OR deepfake OR deepfakes OR “predatory behavior” OR “predatory behaviors” OR predator OR predators OR predatorial OR cyberpredator OR cyberpredators) |  |
| 5 | #1 AND #2 AND #3 AND #4 | 48 |
| 6 | #5 AND **Applied filters: Scholarly Journals** | 31 |
